# Bites of Insects

**Published:** 1857-02

**Authors:** 


### BITES OF INSECTS.

APPLY spirits of hartshorn to them as soon as possible, and almost instantaneous and permanent relief will be given. Reason—the poison of insects, spiders, reptiles, and the like, is an acid; the hartshorn is an alkali, and neutralizes the poison. Any other alkali will answer, but hartshorn is named because people are familiar with it, and it is found in almost every house. A good substitute is a handful of wood ashes thrown into a teacup of hot water, stirred, and as soon as settled, apply the liquid—which is common ley, used for making soap—with a soft rag.

Barnabas Sanders, of Cambridgeport, Mass., was bitten by a spider, several years ago, and on the seventh day, in great agony, expired. Recently a young man in Cincinnati was bitten by a spider, and died in a few days. The celebrated organist of Grace Church, New York, was bitten by a spider, a few weeks since, at twilight, while walking in the garden. Inflammation, pain, and swelling came on, and there appeared a hard tumor, of the size of a goose-egg. In three days the whole glandular system was affected. Singular looking swellings appeared in various parts of the body; one, as large as the egg of a canary bird, appeared under the left eye, and the whole features were distorted and haggard. Dr. Bruce, of Boston, was called, and by his care the patient was eventually restored.

Most probably the prompt and free application of hartshorn, or other strong alkali, would have prevented all subsequent trouble.

The question arises, are the bites of some spiders hurtful and others innocuous, or is the mischief which occasionally results, owing to the state of the blood of the person? We are inclined to the belief that it is the latter. Men have died from the sting of a wasp or bee, but many persons have been stung by bees, wasps, yellow-jackets, hornets, or by whatever other names they are called, and suffered no material injury. We have known men to "*bark their shins*"—to scrape the skin on the fore part of the leg—by a misstep on getting into a carriage, or by striking it against some hard substance inadvertently, the parts became angry-looking, red, hot, and painful, with subsequent symptoms, which have required months of attention to remove, and in some cases ending in death. We know that persons who are young and in vigorous health meet with such mishaps, but let them alone and they get well of themselves. In the latter case the blood is pure and healthy, and full of life; in the former it is diseased. The blood of a person who drinks spirits or beer habitually, is always in this diseased condition; and it is notorious, that even slight abrasions on the hands of such arc not only very hard to cure, but are dangerous. This is owing to the condition of the blood. We think, therefore, that it is a legitimate deduction, that the state of the blood is the cause of the mischief in such insect bites as are followed by serious symptoms. The conclusion then, which we come to in reference to the whole subject of bites by insects, flies, reptiles, and the like, is—

*First.* Apply hartshorn, or some other alkali, as soon as possible, and freely, with a linen rag.

*Second.* Secure a healthy condition of the blood by lives of regularity of bodily habits, of temperance in the indulgence of all appetites, and of moderate industries in the laudable callings of human life.

But it is proper, having with it something of the fearful, to contemplate the brittle thread on which is hung the life of any one who habitually uses any of the brandies and beers of the times. His very existence is at the mercy of the slightest accident; at the mercy of any of the millions of insects which throng the air, which dangle from the trees, or hang upon the rose-bush; for him, death waits everywhere.

